# Three-Dimensional Printing of Hydrogels for Flexible Sensors: A Review

**DOI:** 10.3390/gels10030187

**Published:** 2024-03-08

**Authors:** Suhail Ayoub Khan, Hamza Ahmad, Guoyin Zhu, Huan Pang, Yizhou Zhang

**Affiliations:** 1 School of Chemistry and Chemical Engineering, Yangzhou University, Yangzhou 225009, China; 2 Institute of Advanced Materials and Flexible Electronics (IAMFE), School of Chemistry and Materials Science, Nanjing University of Information Science and Technology, Nanjing 210044, China; iamhamza012@gmail.com (H.A.); gyzhu@nuist.edu.cn (G.Z.); 3 State Key Laboratory of Organic Electronics and Information Displays & Institute of Advanced Materials (IAM), Nanjing University of Posts & Telecommunications, 9 Wenyuan Road, Nanjing 210023, China

**Keywords:** hydrogels, 3D printing, flexible sensors, E-skin

## Abstract

The remarkable flexibility and heightened sensitivity of flexible sensors have drawn significant attention, setting them apart from traditional sensor technology. Within this domain, hydrogels—3D crosslinked networks of hydrophilic polymers—emerge as a leading material for the new generation of flexible sensors, thanks to their unique material properties. These include structural versatility, which imparts traits like adhesiveness and self-healing capabilities. Traditional templating-based methods fall short of tailor-made applications in crafting flexible sensors. In contrast, 3D printing technology stands out with its superior fabrication precision, cost-effectiveness, and satisfactory production efficiency, making it a more suitable approach than templating-based strategies. This review spotlights the latest hydrogel-based flexible sensors developed through 3D printing. It begins by categorizing hydrogels and outlining various 3D-printing techniques. It then focuses on a range of flexible sensors—including those for strain, pressure, pH, temperature, and biosensors—detailing their fabrication methods and applications. Furthermore, it explores the sensing mechanisms and concludes with an analysis of existing challenges and prospects for future research breakthroughs in this field.

## 1. Introduction

Over the past two decades, there have been significant strides in the field of flexible electronics, finding applications in diverse areas such as flexible sensors, soft robotics, energy devices, touch panels, electronic skin, real-time health-monitoring devices, and flexible displays. Flexible electronics encompass circuits and electronic components capable of maintaining functionality even when bent, rolled, folded, or stretched [1,2,3]. Their unique properties of portability, lightweight design, and stretchability, coupled with miniaturization compared to traditional counterparts, have spurred interdisciplinary research across fields ranging from biology to material sciences [4,5,6].

From a materials perspective, flexible electronics usually incorporate various material systems into the design, such as conductive/semiconductive functional materials and insulating/encapsulating matrix materials [7]. These materials can be classified into two categories based on their composition: organic and inorganic flexible electronics [8]. The former is a build-up of polymers like conductive polymers (CPs), while the latter constitutes materials like silicon and thin metal films. In both classes and their hybrid systems, plastic films and soft elastomers often serve as matrix materials. Nowadays, the main approach to designing flexible electronic devices is the integration of inorganic or organic materials such as nanoparticles/nanowires, metal oxides, and conductive polymers into flexible substrates [9]. However, conventional conductive functional materials exhibit various drawbacks, including elevated cost, intrinsic stiffness, poor biocompatibility/mechanical properties, and subpar interface-bonding qualities [10]. Therefore, there is a considerable desire to develop novel materials that address these shortcomings and increase their practical application.

Hydrogels exhibit three-dimensional network topologies, inherent softness, extreme pliability, stretchability, biodegradability/biocompatibility, self-healing, and the ability to absorb huge amounts of water. These characteristics make them well-suited for applications in drug delivery, regenerative medicine, water treatment, and mechanical/biofunctional engineering [11,12]. Furthermore, hydrogels respond precisely to external stimuli like pH, temperature, and stress/strain, making them particularly applicable in flexible electronics [13]. The rapid development of hydrogel applications in smart devices, soft robotics, healthcare, and human–machine interactions has gained momentum recently [14]. For utilization in flexible electronics, hydrogels must possess electrical conductivity. Various fabrication procedures have been proposed to impart this property, leading to the development of numerous conductive hydrogels based on ion or electron transport [15].

To comprehend the practicality of electrically conductive hydrogels in flexible electronics, conventional casting, and templating techniques are commonly employed to generate suitable structures [16]. However, the reliance on molds, dies, or masks in these procedures restricts customization, resulting in the production of only basic 3D structures [17]. Additionally, the gelation method entails an enormous amount of liquid precursors for in situ solidification leading to a substantial loss of energy and resources. Furthermore, it has been demonstrated that the performance of flexible electronics can be enhanced by meticulous structural design, such as incorporating hierarchical inner architectures at the micro- and nanoscale [18]. To address these problems, 3D printing is viewed as the most effective method for engineering complex and precise 3D structures [19]. The utilization of 3D printing allows for the precise fabrication of stretchable hydrogels with intricate 3D designs, structural combinations, and superior functionalities. However, there are a few obstacles like material property (viscosity, thixotropy), crosslinking methods, multi-material printing, cost efficacy, print resolution, and structural integrity, which need to be addressed for the advancement of the competencies of 3D printing with gels [20].

Thanks to modern technology advancements, traditional sensors, with their inherent limitations, are gradually being replaced by flexible sensors across various domains. Flexible sensors are designed from materials that endow them with the ability to bend or fold with ease [21]. These sensors exhibit exceptional accuracy and reliability in detecting physical quantities, respond quickly, and find applications across a diverse range of fields [22]. However, the applicability of flexible sensors created via conventional techniques is often constrained due to challenges related to cost, manufacturability, and design freedom [23]. However, the advent and evolution of 3D printing have revolutionized the fabrication and utilization of flexible sensors [24]. Recently, many breakthroughs have occurred in the intersection of 3D printing and hydrogels for flexible sensors. Despite its importance, the integration of these two technologies is still in its infancy. Thus, to guide future research efforts, it is crucial to present an overview of the state of the field. Many reviews that summarize the most recent developments in the field of 3D printing concerning robust hydrogels have been published [25,26]. Their thorough investigation makes a substantial contribution to the rapidly developing field of 3D printing by offering insightful information on the development of durable hydrogel materials and their uses. However, 3D-printed hydrogels for flexible sensors have not been overviewed substantially. This review classifies hydrogels, explores various 3D-printing methodologies, and outlines the fabrication of hydrogel-based flexible sensors using 3D-printing technology and sensing mechanisms. Additionally, the review discusses opinions and perspectives on the future directions of this intriguing field.

## 2. The 3D Printing of Hydrogels

The term “3D printing” refers to a range of technologies enabling the layer-wise construction of three-dimensional parts from computer-designed models [27]. The notion of 3D printing originated in the 1980s with the development of stereolithography (SLA), which utilizes a UV laser to harden layers of liquid photopolymer resin. Consequently, in the late 1990s, other 3D-printing approaches like fused deposition modeling (FDM) and selective laser sintering (SLS) were established that endowed the fabrication of 3D objects via layered fashion from digital designs [28]. By introducing hydrogels into the 3D-printing process in the early 2010s, developments in the field made it possible to create structures made of hydrogels with complex shapes. Typically, 3D printing has some characteristic advantages over traditional approaches like design flexibility, rapid prototyping, reduction in materials waste, on-demand production, and being economically viable. However, it suffers from the flaws of surface resolution, limited material selectivity, and production speed, which impedes its utilization in different fields [29]. The 3D printing of hydrogels can be divided into three categories: material jetting, material extrusion, and vat photopolymerization, depending on how the hydrogel components are formed into three-dimensional structures.

### 2.1. Material Jetting

#### Inkjet-Printing Method

**Figure 1:**
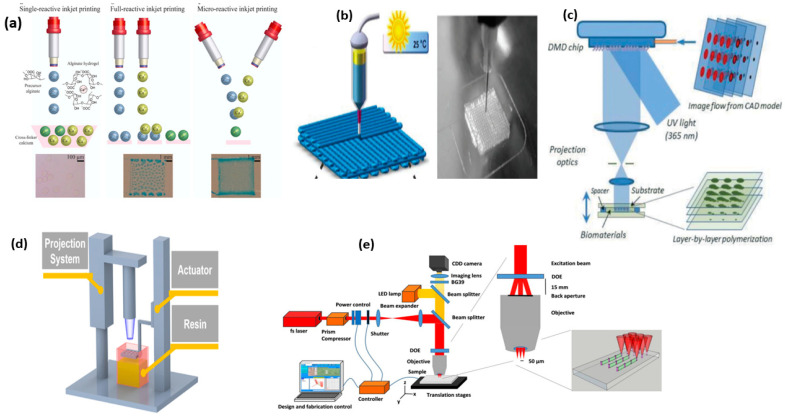
Representation of 3D printing by various approaches, inkjet (**a**) reproduced with permission [32]. Copyright © 2020 American Chemical Society, Washington, DC. Direct ink (**b**) reproduced with permission [40]. Copyright © 2019 Royal Society of Chemistry, London. Stereolithography (**c**) reproduced with permission [45]. Copyright © 2014 Royal Society of Chemistry. Digital light processing (**d**) reproduced with permission [48]. Copyright © 2023 American Chemical Society, and two-photon polymerization (**e**) reproduced with permission [52]. Copyright © 2023 WILEY-VCH Verlag GmbH & Co. KGaA, Weinheim, Germany.

Inkjet printing is considered appropriate for multi-material processing since it can work with many printheads, offers high resolution, operates without physical contact, and has easily scalable processing capabilities [30]. Inkjet printing utilizes low-viscosity fluids and operates by shooting liquid ink droplets out of a nozzle and onto the target object [31,32] by a driving force (Figure 1a). The key characteristics to consider while choosing an ink are its fluid characteristics, including viscosity, density, and surface tension, as these attributes determine the material’s ability to be jetted and affect the size and shape of the droplets deposited. The ink’s viscosity should be sufficiently low to allow for refilling the ink reservoir in approximately 100 milliseconds and expelling a drop from the nozzle using a transient pressure pulse. The surface tension needs to be sufficient to avoid dripping from the nozzle but also low enough to allow the droplet to detach from the nozzle [33]. For inkjet printing, the recommended viscosity ranges from 1 to 25 mPa·s, whereas the surface tension should be between 25 and 50 mNm^−1^. These values may differ depending on the specific printer [34]. In hydrogel printing through inkjet technology, the reactive inkjet-printing technique is commonly employed to address challenges like viscosity, agglomeration, and polymerization flaws. This method involves the successive deposition of precursors and crosslinkers from printheads, reacting at specific substrate locations to create the desired pattern. Reactive inkjet printing is favored for hydrogel production, especially when additional crosslinkers and initiators are needed for the transition from a liquid to solid state [35]. A recent study demonstrated the utilization of thermal inkjet printheads and aquatic calcium ions as inks for printing on hydrogel surfaces, creating a pressure sensor with efficient compression strength and a steady shift in capacitance [36]. Given that inkjet printing is limited to low-viscosity materials, further exploration is crucial to expand the applicability of this method.

### 2.2. Material Extrusion

#### Direct-Ink-Writing (DIW) Method

Owing to the economic viability and facile printing technique, extrusion-based printing is one of the most competent choices for designing 3D structures. In this method, the materials are initially extruded through a nozzle using a mechanical force after which the deposited polymer solidifies via processes such as chemical crosslinking, recovery of noncovalent bonds, crystallization, or chain rearrangement [37]. Following the completion of a single layer, the build platform lowers or the extrusion head travels upward to deposit the subsequent layer [38]. The formation of hydrogel-based 3D structures is potentially accomplished by the direct ink approach of the extrusion process. The direct ink method employs extrusion to propel ink from the nozzle using external force, followed by a curing process to solidify the as-printed structure [39]. Recognized as one of the best techniques for printing hydrogels (Figure 1b), this method offers a wide range of material options, high controllability, and ease of execution [40]. In a recent study, polyvinyl alcohol and polyacrylamide were utilized to create an ionic conductive, fluid hydrogel material. At room temperature, this material easily extrudes due to its fluidity. Subsequently, the crosslinked curing of acrylamide is then finished when exposed to UV light. Lastly, the material was immersed in a borax solution to yield a strong conductive flexible material [41]. Typically, the viscosity of the ink for the direct ink method process needs to fall within the range of 10^−1^ to 10^3^ Pa S to facilitate the printing process. This limitation makes it challenging to use diluted hydrogel inks as printing materials. However, a study verified the direct ink printing of dilute hydrogel inks (10^−3^ Pa S) via facilitating substrate and ink interaction [42], opening the doors to printing hydrogels via direct ink methodology. Further, indirect bioprinting, coaxial nozzle printing, and the addition of nanomaterials in bioinks could improve the printability of hydrogels printed via the extrusion approach [43]. 

### 2.3. Vat Photopolymerization

#### 2.3.1. Stereolithography Method

Unlike inkjet and extrusion 3D printing, which require nozzles, vat photopolymerization uses an irradiation light source to provide the necessary energy to cause photopolymerization, harden a photosensitive polymer ink, and enable layer-by-layer printing. The most prevalent vat photopolymerization methods include stereolithography (SLA), digital light processing (DLP), and two-photon polymerization (TPP). The primary distinctions among these 3D printing systems lie in the light source and imaging system, whereas the control and stepping mechanisms are comparable [44]. 

Stereolithography (SLA), a light-based 3D-printing technique employing vat polymerization, involves exposure to laser or UV light, causing it to solidify and form layers in a layered fashion (Figure 1c). The quality of printing in SLA is dependent on the precision of the laser system [45,46]. Owing to the laser system’s focusing ability, SLA allows the formation of large and intricate objects with micrometer-scale structural topographies. Moreover, bulk printing along with affordability, good flexibility, less complexity, and ease of use renders SLA as a superior approach to printing hydrogels. An ionic composite hydrogel was fabricated via SLA, producing a 3D material with commendable mechanical strength, notable conductivity, and osmotically driven actuation [47]. Despite the potential benefits of SLA, its reliance on photopolymerizable resin poses a significant limitation. Additionally, the resins ought to exhibit light transparency, superior rheological performance, and low viscoelasticity. Also, SLAs suffer from low printing speeds due to the generation of one voxel at a time using a point-wise single photon.

#### 2.3.2. Digital-Light-Processing Method

Digital light processing (DLP) operates on the principle of vat polymerization, similar to SLA. However, unlike SLA, which employs a spotlight source to cure the material (Figure 1d), DLP relies on micromirrors or liquid crystal display for the curing process, using a 2D cross-sectional light projection [48,49]. This results in significantly faster printing speeds with high output and resolution compared to SLA. Additionally, the DLP technique is not constrained by the high transparency of resin, allowing for greater flexibility in the printing material selection process. Based on these benefits, a variety of hydrogels are printed using DLP. A study revealed the design of a hydrogel sensor based on DLP. The biodegradable hydrogel sensor depicted a higher gauge factor (1.5–7.2) within a strain range of 10–100% enabling it to be used for tracking a range of human activities [50]. A self-healing hydrogel was fabricated using DLP, and this printed material could be easily restored to the original state freely at room temperature in the absence of any outside stimulation [51].

#### 2.3.3. Two-Photon Polymerization Method

The two-photon polymerization (TPP) method demonstrates the ability to craft intricate 3D structures with nanoscale resolution using a near-infrared femtosecond laser (Figure 1e) capable of inducing the polymerization of photosensitive materials [52,53]. The ultra-fast laser generates reactive species that trigger the polymerization of acrylate and epoxy monomers, making the effectiveness of photoinitiators crucial for the quality of the TPP-printed structures. The TPP approach can print 3D objects with better precision by carefully regulating the printing parameters, such as laser power intensity, sensitive photoinitiators, scanning rate, etc. Despite the capability of TPP to produce precise assemblies with ultra-high spatial resolution, its high price, compact size, slow printing speed, and post-treatment requirement limit its widespread usage. 

## 3. Classification of Conductive Hydrogels

Considering the innate insulating nature of hydrogels, it becomes critical to alter them to increase their conductivity [54]. These modified hydrogels can be classified into two types: electrical conductivity hydrogels (ECHs) and ion conductivity hydrogels (ICHs) [55]. In the former, conductivity is attained via the utilization of a conductive material, either by filling or coating. Furthermore, hydrogels synthesized from polymers with a π system exhibit electronic conductivity [56]. In the latter type, conductivity is realized through the formation of ion channels for which enough free electrons are made available in the hydrogel [57]. Among versatile designing methods for producing intricate geometries and customized features of ECHs and ICHs, SLA and DIW have been demonstrated as valuable methods to fabricate these hydrogels. The printability concentration range of precursors is typically from 10–30% for ECHs and, for ICHs, it is from 2 wt% to tens of wt% [58].

### 3.1. Electron-Conducting Hydrogels (ECHs)

Electron-conducting hydrogels are a class of advanced materials that can conduct electrons. They owe the conductivity to the freely flowing electrons in metals and the delocalized π electrons in other materials. Typically, these ECHs are classified into three categories based on the material used as an electron-conducting component, which include metallic ECHs (containing metal nanofillers Au, Ag, Cu, nanowires/nanoparticles), carbon-based ECHs (graphene, carbon nanoparticles, CNTs), and conducting polymer ECHs (polypyrrole, polyaniline, polythiophene, poly (phenylene vinylene)) [59]. 

**Figure 2:**
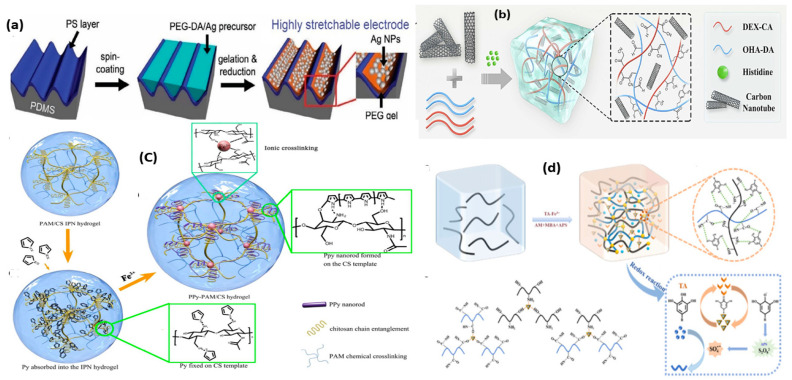
Schematic representation of ECHs using in situ polymerization, in which metal ions are reduced to metal nanoparticles (**a**), reproduced with permission [63]. Copyright © 2011 WILEY-VCH Verlag GmbH & Co. KGaA, Weinheim. Fabrication of conductive CNT-based hydrogels (**b**), reproduced with permission [66]. Copyright © 2023 Elsevier Ltd, Amsterdam. Illustration of polypyrrole-based composited conductive hydrogel, in situ polymerization, and chain entanglement (**c**) reproduced with permission [73]. Copyright © 2018, American Chemical Society. Schematic representation of ICHs, possible mechanism, and redox process to endorse free radical polymerization (**d**) reproduced with permission [74]. Copyright © 2022 Elsevier B.V.

Owing to the distinctive traits of metallic nanoparticles, such as high electrical conductivity/large specific surface area and magnetic, optical, and catalytic qualities, they have been employed extensively in the fabrication of ECHs [60]. Concerning the stability perquisites of metallic nanoparticles, the frequently used metals are gold and silver. Gold is chosen for its exceptional electron transport capacity, while silver boasts the maximum electrical conductivity. A study demonstrated the anchoring of Ag^+^ with the deprotonated-COOH group of the hydrogel matrix, followed by the reduction of ions to Ag nanoparticles, resulting in an ECH with electrical properties responsive to pH [61]. The deposition of Ag nanoparticles via in situ reduction into a polydopamine-based hydrogel was achieved, yielding a material with high piezoresistive sensitivity (up to 9.34 MPa^−1^). This capability allows simultaneous monitoring of minute pressure signals and massive body motions [62]. The 3D printing showed the photoreduction and photopolymerization of Ag^+^ and polyethylene glycol diacrylate, respectively (Figure 2a), combining them into a single technique for the rapid creation of intricate, electrically functioning 3D objects [63]. While ECHs based on metal nanoparticles often have higher conductivities, a major concern is the uneven distribution of the material, limiting their usage in 3D printing, which requires material consistency. Thus, further research is essential to address this challenge in metal-based ECHs.

Carbon-based materials offer fresh prospects for constructing ECHs within polymer matrices. These networks not only create channels for electron transport via the π-conjugated structure but likewise improve the mechanical characteristics of the materials because of their large surface area and numerous surface functionalities [64]. Typically, carbon nanotubes (CNTs) and graphene have been the most extensively employed kinds of carbon materials as conductive fillers for the creation of conductive hydrogels [65]. Ding et al. introduced CNTs into dopamine-modified oxidized hyaluronic acid and cyanoacetate group-functionalized dextran (DEX-CA) to fabricate a hydrogel (Figure 2b) with high sensitivity, good mechanical strength, self-healing capability, and on-demand removability for wearable electronics [66]. The wrapping of CNTs with alginate demonstrated a synthesized material with a tensile strength of 332.9 kPa and electrical conductivity of 2.76 S m^−1^ [67]. Additionally, a study utilized graphene-carboxy sheets as reinforcement in the fabrication of gelatin methacrylate (GelMA), polypyrrole, and alginate-based hydrogel. The electrical conductivity reached 0.124 S m^−1^ and the tensile strength was 133 kPa [68]. Due to their huge specific surface area, superior mechanical properties, and delocalized π bonds, carbon material provides several benefits in the development of ECHs. However, in the realm of 3D printing, challenges such as the inherent blackness/opacity of carbon materials and the optimization of CNT dispersion significantly constrain their usage, prompting the exploration of new avenues.

Of late, there has been a mounting interest in using conducting polymers to create ECHs. These polymers offer some benefits over other conducting materials, including solubility, biocompatibility, flexibility, and adaptable electronic conductivity [69]. The conductive polymer-based hydrogels did not exhibit structural differences between the hydrogel matrix and conductive materials. This absence of a strong interface effect, common in organic and inorganic materials combinations, provides an advantage by enhancing both electrical conductivity and mechanical properties [70]. Currently, conductive polymers, such as polyaniline (PANI), polythiophene (PTh), polypyrrole (PPy), and poly(3,4-ethylene-dioxythiophene) (PEDOT), are typically utilized to prepare conductive hydrogels [71]. A study demonstrated the fabrication of a hydrogel based on a polyaniline/poly (4-styrene sulfonic acid) (PANI/PSS) network using -ureido-4[1H]-pyrimidine as a crosslinker [72]. The hydrogen bonding in this hydrogel imparts flexibility, allowing it to be molded into various shapes and recover within 30 seconds. A new approach by Gan et al. [73] involved the in-situ polymerization of PPy in a hydrogel matrix using chitosan as a template (Figure 2c). The resulting conductive hydrogels demonstrated exceptional mechanical properties and improved electrochemical attributes. Conductive polymers generate electrical conductivity by allowing delocalized π electrons to flow freely, creating an electronic channel. When coupled with a hydrogel matrix, conducting polymers offer distinct benefits over metal nanoparticles and carbon materials. Nevertheless, many conductive polymers are inherently brittle and stiff. To improve mechanical properties for flexible electronics applications, it is frequently necessary to integrate them with other flexible materials or media capable of forming noncovalent connections.

### 3.2. Ion-Conducting Hydrogels (ICHs)

Hydrogels can allow the free movement of water molecules across the polymeric network. The inherent high ion mobility in water makes it possible to develop ICHs, typically created by the direct introduction of free ions (Figure 2d) [74]. In general, three types of ICHs, acidic ICHs (HCl, H_2_SO_4_, H_3_PO_4_) basic ICHs (NaCl, KCl, LiCl, FeCl_3_, CaCO_3_/CaCl_2_,), and ionic liquid ICHs (1-ethyl-3-methylimidazoliumchloride), are classified based on the material used that can generate free ions in water and can confer ion conductivity through mechanisms such as ion hopping, segment motion, or a vehicle mechanism [75]. The ICHs exhibit several advantages and superior characteristics compared to ECHs, which broaden the range of possibilities for them in flexible electronics. Their transparent surface renders them competent in the photoelectronic field, while the nonutilization of fillers makes them benign, making them beneficial for the surveillance of human health. Additionally, the presence of free ions allows ICHs to form an electric double layer on surfaces that are either rich or lacking in electrons and have the potential to act as electrolytes for solid batteries and perform a key role in actuators [76]. Nevertheless, ICHs heavily rely on ionic aqueous solutions to produce the ion migration route. The ICHs containing aqueous solutions are more susceptible to temperature variations and, below a specific point, the hydrogel will freeze and weaken its mechanical structure and at higher temperatures loss of ionic conductivity due to water evaporation is perceived [77]. This sensitivity of ICHs to temperature was utilized by Pang et al. who designed an ICH in which PNIPAAm was introduced, resulting in quick volume shrinkage and alteration in electrical signals as the temperature reaches a certain point. Further, the synthesized hydrogel exhibits outstanding transparency and good conductivity (0.79 S m^−1^) [78]. Although ICHs show a myriad of advantages, the evaporation problem, low mechanical properties, and paucity of self-adhesive properties restrict their usage in many specific fields and thus research is needed to mask the flaws to make them competent in each arena.

## 4. Sensing Mechanism

**Figure 3:**
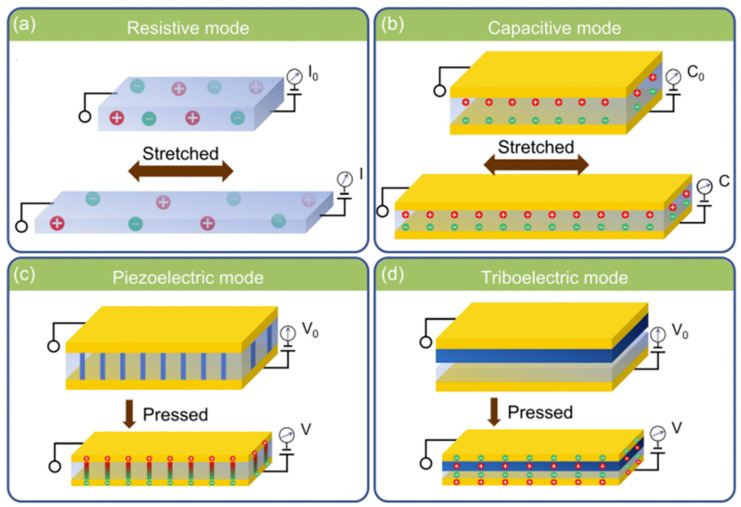
Schematic representation of sensing mechanism of hydrogel-based sensors, piezoresistive (**a**), capacitive (**b**), piezoelectric (**c**), and triboelectric (**d**). Reproduced with permission [79]. Copyright © 2023 WILEY-VCH Verlag GmbH & Co. KGaA, Weinheim.

Hydrogel-based sensors exhibit sensing characteristics mainly via piezoresistive, capacitive, piezoelectric, and triboelectric mechanisms [79] (Figure 3). Among these, piezoresistive sensing has gained prominence due to its facile fabrication and high-pressure sensitivity [80]. The resistance in piezoresistive sensors is defined as R = ρ × (L/A) where R, ρ, L, and A represent resistance, resistivity, length, and cross-sectional area of the material, respectively. An increase in resistivity and length correlates with a rise in resistance, while an increment in cross-sectional area results in a decrease. Hydrogels with conducting nanofillers, when subjected to pressure, create an electron transmission path, altering the resistance [81]. Concerning conductive polymer hydrogels and ionic conductive hydrogels, modifications to the internal pore structure brought on by external pressure are the main causes of variations in resistance levels. In the conductive polymer hydrogel, compression leads to pore contact, shifting the conduction channel and affecting resistance. Ionic hydrogels, which depend on the unhindered flow of ions, may also respond to pressure-induced pore evolution [82]. The capacitive sensing mechanism relies on the dielectric layer’s deformation capacity along with the conductivity of the two electrodes. On applying external pressure, the sensor’s electrodes come closer, resulting in a discernible electrical signal indicating the enhancement in capacitance [83]. In the case of flexible hydrogels, electrical conductivity can be accomplished through the use of conductive polymers or the adoption of an ionic solution that provides the conductive pathways, fulfilling the necessity for electrode materials for capacitive sensors [84]. This ensures electrode conductivity, enabling superior structural fusion with the dielectric layer and mitigating the interface’s impact on mechanical attributes. The triboelectric mechanism involves the creation of a nanogenerator that relies on the self-power attained via the coupling of triboelectric and electrostatic induction [85]. When materials with high electronegativity undergo friction, opposing electric charges are produced. To create a self-power supply, the friction material is then connected to two electrodes via electrostatic induction. In the piezoelectric sensing mechanism, a polarizable charge is generated inside the material on the application of pressure [86]. The polarized charge density rises in direct proportion to the upsurge in outside pressure. There is a generation of current between the electrodes when an external load is connected. The change in current is precisely proportional to the applied pressure, allowing the sensing behavior of the material.

## 5. Application of 3D-Printed Hydrogels in Flexible Sensors

Lately, flexible sensors have garnered significant attention because of their potential application in a myriad of fields including wearable technology, artificial skin, and soft robotics. These flexible sensors possess the ability to detect and convert environmental stimuli into easily interpretable signals. The fundamental elements of flexible sensors consist of active materials electrodes, substrates, and dielectrics [87]. A multitude of flexible sensors have been constructed using soft materials. Among them, hydrogel-based flexible sensors stand out for their flexibility, ease of incorporation of functional moieties, biocompatibility, and high-water content which enhances their ability to mimic natural tissues. However, their lower mechanical strength and conductivity than traditional sensors along with shrinkage/swelling issues restrict their utilization [88]. Thus, while designing the sensors these parameters are taken into consideration as the amalgamation of 3D printing and hydrogels holds promise for creating more intricate architectures for flexible sensors. 

### 5.1. Flexible Strain Sensors

**Figure 4:**
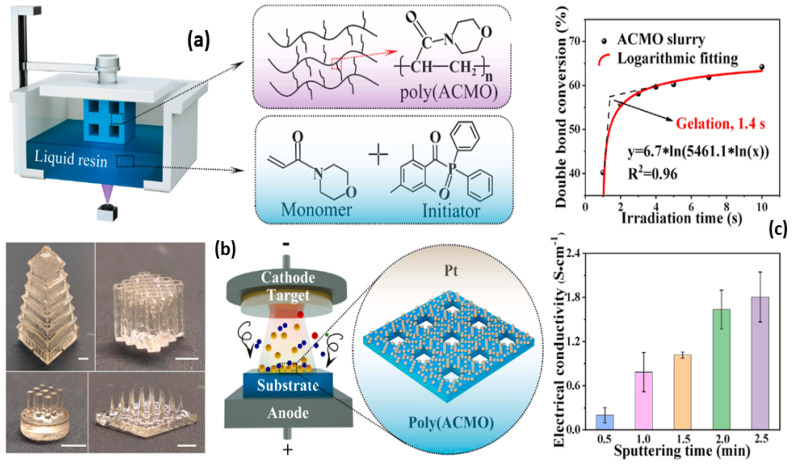
Digital-light-processing approach for the fabrication of hydrogel strain sensor, kinetics of the ACMO slurry (**a**), digital photographs of 3D structures, representation of ion sputtering system (**b**), and electrical conductivity of poly (ACMO) components with various sputtering times (**c**). Reproduced with permission [91]. Copyright © 2022 Published by Elsevier Ltd.

The prerequisite of wearable strain sensors is their ability to monitor the movements of the body’s targeted organs continually and precisely, regardless of the intensity of the activity. Flexible strain sensors are distinct from traditional silicon-based strain sensors in that they are made of flexible and stretchable material [89]. Various approaches to create multiplex configurations in strain sensors have been accomplished via atomic deposition, photolithography, spraying, and sputtering. Nevertheless, these procedures, due to their time inefficiency and wastage of material, have limited commercialization [90]. The 3D-printing technology offers a potential way to produce flexible strain sensors because of its varied raw material selection, precise structural design, and streamlined preparation procedure. Guo et al. fabricated an electrically conductive and degradable poly (ACMO)/pt hydrogel strain sensor [91] via DLP 3D printing (Table 1). The liquid resin was produced via the blending of monomer N-acryloyl morpholine (ACMO) and the photosensitizer diphenyl (2,4,6-trimethylbenzoyl) phosphine oxide (Figure 4a). The gelation was achieved within 14 sec, implying a swift polymerization. The DLP technique makes it easy to fabricate the ACMO suspension to create intricate structures (Figure 4b). After printing, the conductivity was obtained by Pt sputtering and the conductivity reached 1.6 S cm^−1^ at 2 min of sputtering (Figure 4c). The poly (ACMO)/Pt hydrogel sensor demonstrated excellent cyclic stability, a high gauge factor (GF) of 1.5 and 7.2 at 0–10 and 10–100% strain, and outstanding feasibility to monitor a range of human activities. Furthermore, it depicted a complete degradation when placed in gastric juice (5 h) and soil (10 days). Li et al. utilized κ-carrageenan/AAm as an ink to print intricate 3D structures [92].

**Figure 5:**
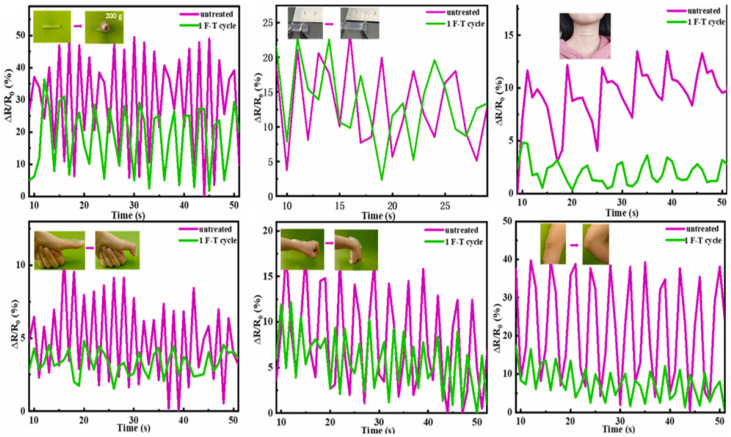
Real-time variations in relative resistance during external compression, stretching, deep breathing, finger flexion, wrist movements, and changes in elbow motion using the B-PVA/kC_1_ hydrogel sensor. Reproduced with permission [93]. Copyright © 2023 Elsevier B.V.

The 3D-printed strain sensor based on κ-carrageenan/Aam double network hydrogel demonstrated better strain sensitivity, having a GF of 0.63 at 1000% strain, making it ideal in human motion recognition. A borax, PVA, and κ-carrageenan (B-PVA/kC) hydrogel was printed into different 3D structures to fulfill the demand for pliable strain sensors in various forms [93]. The B-PVA/kC sensor precisely monitored large movements (fingers, wrist, elbow) as well as small movements (deep breathing) (Figure 5). The hydrogel-based flexible sensor demonstrated better sensing potential after 1F-T cycles, and the GF is 0.42 over a 0–50% strain range. 

**Figure 6:**
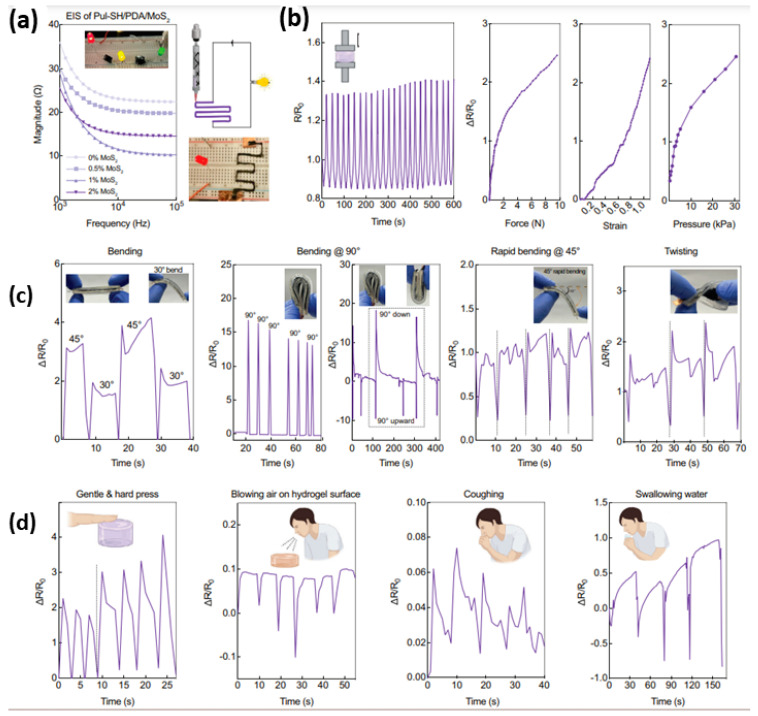
Electrical conductivity of Pul-SH/PDA/MoS_2_ hydrogel sensor and its utility as an electronic skin for strain and pressure sensing (**a**). Electrical impedance spectroscopy plots (**b**). Relative change in resistance values (ΔR/R0) of the hydrogel Pul-SH/PDA/MoS_2_ in reaction to external force, strain, and pressure variations and numerous cycles of compression (25 cycles), showing linearity in response (**c**). Vibrations recorded from the two different acts (**d**). Reproduced with permission [95]. Copyright © 2024 Wiley-VCH GmbH.

Electronic skin (E-skin) has the potential to revolutionize wearable technology, robotics, and healthcare by imitating the elasticity and stretchability of human skin, coupled with advanced sensing capabilities [94]. However, producing robust materials that are flexible like skin, including biosensing capabilities, and employing cutting-edge fabrication methods for wearable or implantable applications are only a few of the major obstacles in the development of E-skin. Roy et al. developed a 3D-printed elastomeric hydrogel via triple crosslinking involving defect-driven gelation, Michael addition, and ionic crosslinking [95]. The crosslinked hydrogel was linked to the circuit and demonstrated successful light-emitting diode (LED) bulb lighting (Figure 6a), indicating the potential usage of the hydrogel in making 3D-printed electronic skin. 

Following compression, the sensor exhibited a subtle fluctuation in resistance change over time during both the loading and unloading phases of the compression cycle (Figure 6b). Clearly, the E-skin responded precisely, with high sensitivity and reproducibility of strain variations. For real-time applications, a wearable E-skin was developed by encapsulating 3D-printed hydrogels between two thin layers of polydimethylsiloxane (PDMS). This wearable device was designed to track a variety of human movements, including twisting and bending. The wearable device was bent at 30°, 45°, and 90°, and it was twisted repeatedly to quantify the variations in resistance (Figure 6c). These findings unequivocally show the promise of employing the pullulan hydrogel as an E-skin to detect various human patterns and motions. Furthermore, the hydrogel sensor’s sensitivity to varying pressure magnitudes was demonstrated by its ability to respond appropriately to both light and heavy finger presses (Figure 6d). Amazingly, the hydrogels’ ability to sense air blowing on their surface was demonstrated by a shift in resistance.

**Figure 7:**
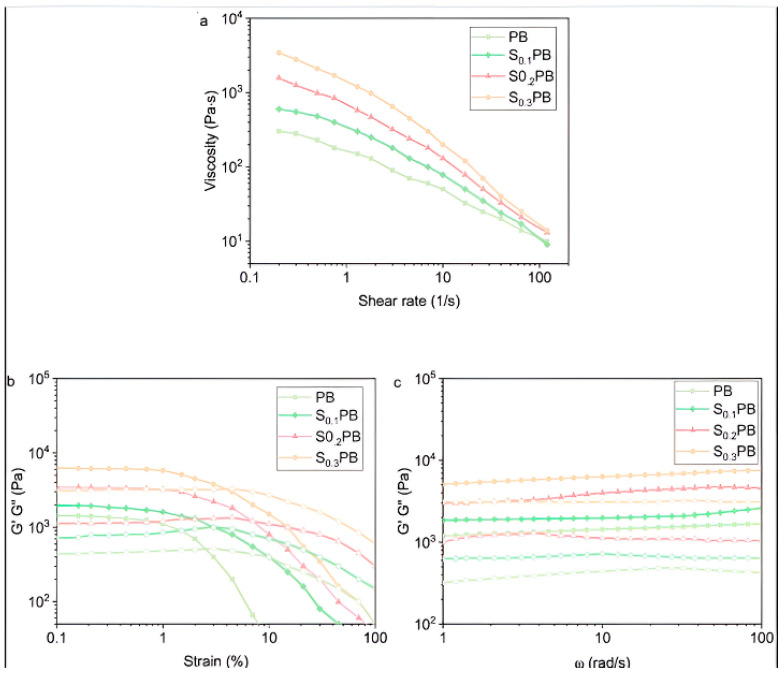
Shear rate as a function of viscosity (**a**). Strain sweep at a fixed frequency of 10 rad⋅s^–1^ (**b**). Oscillatory frequency sweep at a fixed shear strain of 0.1%. G′ (**c**). Reproduced with permission [98]. Copyright © 2022 Elsevier B.V.

A bioinspired 3D-printable stretching hydrogel as a wearable E-skin sensor was developed by Wei et al. [96]. They incorporated carbon nanotubes in calcium ion chelate with polyacrylic acid and sodium alginate. A 3D extrusion printer was employed to print the hydrogel on dielectric tape connected to an electrode, and an E-skin strain sensor was developed that responded steadily and sensitively to external stimuli like breathing, knee flexion, and finger flexion. The E-skin sensor demonstrated a GF of 6.29 and 1.25 kPa^−1^ in resistance and capacitance mode, respectively. A study showcased the integration of 3D printing and microfluidics to craft patterned E-skin utilizing conductive hydrogel [97]. The produced E-skin exhibited remarkable flexibility and sensitivity due to the microfibers’ excellent mechanical and electrical performance. These features suggested that the conductive hydrogel microfiber-based E-skin could be effectively used in wearable technology. Polyvinyl alcohol/sodium tetraborate/sodium alginate hydrogel ink with superior mechanical, rheological, and self-healing capabilities was prepared by a one-pot synthesis and then extrusion 3D printing was exploited for the development of a self-healing hydrogel [98]. The shear-thinning characteristic of the hydrogel ink was substantial. With an upsurge in shear rate from 0.2 to 120·s^−1^, the viscosity of hydrogel drops off (3433 to 14 Pa·s), authenticating the adequate flow characteristics of hydrogel ink during extrusion-3D printing (Figure 7a). Sodium alginate enhanced the mechanical properties of the hydrogel as seen by the strain sweep test wherein the hydrogels’ storage modulus (G′) and loss modulus (G″) rise with sodium alginate quantity, illustrating that sodium alginate substantially enhances its mechanical characteristics (Figure 7b). This behavior suggests that sodium alginate is evenly distributed throughout the hydrogel network and establishes hydrogen bonds with PVA and water molecules to reinforce the hydrogel’s network structure. Furthermore, the effect of oscillation frequency on hydrogel (Figure 7c) was examined, which showed that the hydrogel was devoid of any sol–gel transition and G′ > G″. The hydrogel strain sensor has elevated stretchability (>2800% strain), decent conductivity, and sensitivity (GF: 18.56 at 2000% strain), allowing for the tracking of human motions and weak vibrations like breathing and the flexing of the fingers and knees. Feng et al. revealed the fabrication of a double network hydrogel sensor with poly (sulfobetaine-co-acrylic acid)/chitosan-citrate (P(SBMA-co-AAc)/CS-Cit) with anti-fatigue, self-adhesive, highly stretchable, and self-healing characteristics [99]. The hydrogel exhibits strong electrical conductivity (0.11 S·m^−1^), fair sensitivity to extensive strain, great transparency, and outstanding self-healing capabilities (95.4%). In addition to detecting a variety of human activities, including joint bending and swallowing, the hydrogel sensor establishes a steady and dependable relative resistance change during deformation. A 3D-printed polyvinyl alcohol/carboxylated chitosan/sodium alginate/silver nanowire hydrogel was fabricated, which demonstrated admirable recoverability and fatigue resistance owing to the hydrogen binding and ionic interactions. Furthermore, the presence of silver nanowires as fillers makes it detect human motion, small strains, and temperature [100]. 

**Figure 8:**
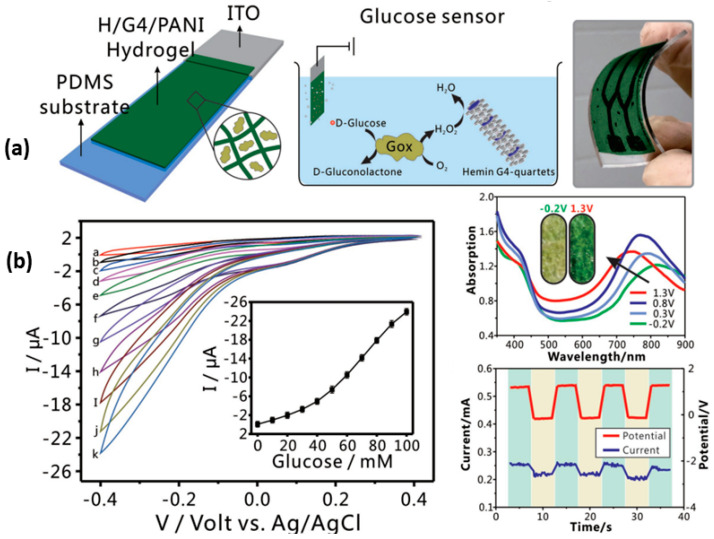
Schematic representation of GOx-loaded H/G4–PANI hydrogel sensor along with its optical images (**a**), cyclic voltammograms, UV–Vis absorption spectra, and potential and chronoamperometry curves (**b**). Reproduced with permission [102]. Copyright © 2018 WILEY-VCH Verlag GmbH & Co. KGaA, Weinheim.

Glucose monitoring has been used for over four decades for diabetes management. Several glucose-monitoring methods, including optical, electrochemical, and impedance spectroscopy, have been investigated and refined. Lately, some 3D-printing techniques have been employed to create wearable, lightweight flexible sensors for measuring and quantifying glucose. Li et al. reported a drop-on-demand inkjet-printing process for the production of a hydrogel-based multiplexed flexible glucose sensor, which gives a real-time measurement of glucose, lactate, and triglycerides with excellent selectivity and sensitivity [101]. Another study revealed that a nanofibrous hydrogel based on guanosine and KB(OH)_4_ has considerable enzyme-like activity, which can be used as ink for the printing of glucose sensors (Figure 8a) [102]. It demonstrated directly injecting an electrochemical electrode, which is advantageous for fabricating a flexible glucose sensor by piling glucose oxidase as represented by corresponding plots (Figure 8b).

### 5.2. Flexible Pressure Sensors

**Figure 9:**
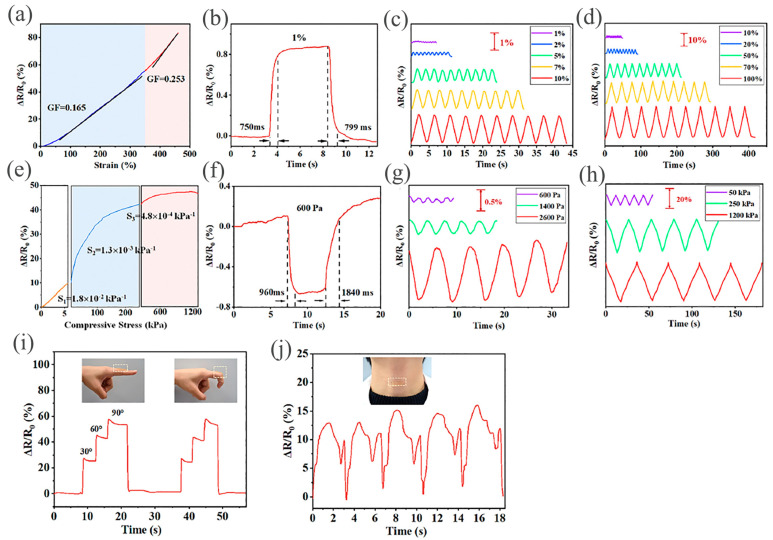
Sensory operations (**a**). Resistance alteration ratio of hydrogel sensor during stretching (**b**). Response time at 1% strain (**c**). Resistance alteration ratios under various strains (**d**). Compressive-pressure-sensing hydrogel sensor (**e**). At 600 Pa, the response time (**f**). Resistance alteration ratio at different compressive stresses (**g**). Resistance alteration ratio changes over 600 cycles (**h**). Resistance alteration when attached to human finger (**i**) and neck (**j**). Reproduced with permission [105]. Copyright © 2023 Royal Society of Chemistry.

Flexible pressure sensors, characterized by outstanding flexibility, sensitivity, and durability, represent a promising avenue for continuous monitoring of human health and activities [103]. They find their usage in applications in wearable technology, soft robots, electronic skin, physical health detection, and artificial intelligence [104]. They can provide crucial information regarding particular requirements that the human body has, both inside and when it comes into contact with the outside world. Zheng et al. fabricated microgel-reinforced double network hydrogels for which polyelectrolyte poly(2-acrylamido-2-methylpro-panesulfonic acid) and acrylic acid were amalgamated to produce inks for 3D printing [105]. The sensor depicted GFs of 0.16 and 0.25 at 0–350 and 350–460% strain, respectively (Figure 9a). The sensor exhibited a swift reaction (750–799 ms) to 1% strain (Figure 9b). Owing to the exceptional mechanical characteristics of the hydrogel-based sensor, precise sensing signals are demonstrated at both small (1–10%) and large (10–100%) strains, respectively (Figure 9c,d). The pressure sensitivity at 0–5 kPa depicted by this sensor was 0.018 kPa^−1^ (Figure 9e), and the response time was 960–1840 ms at a low pressure of 600 Pa (Figure 9f). The hydrogel sensor showed superior sensor applicability for delicate human behaviors as well as heavy ones like walking. A rise in ΔR/R0 was detected with a finger-bending angle in the hydrogel sensor and, when the finger straightens back up, the resistance goes back to its initial value (Figure 9g). Furthermore, the sensor was attached to the neck, which produces steady sensory signals in response to the larynx node vibrating during eating (Figure 9h). A study demonstrated the utilization of DLP to produce a double network ionic conductive hydrogel constituting acrylic acid/acrylamide and MgCl_2_ [106]. The hydrogel’s mechanical characteristics, tunability, and printability (150 μm) make it conceivable to print iontronic pressure sensors conveniently. The iontronic pressure sensor has exceptional stability (200 cycles of pressure loading) and an extensive detection range (26 Pa–70 kPa), in addition to its high sensitivity (0.06 kPa^−1^). All these findings showed that the iontronic sensor could be used to detect human movements and achieve tactile sensation in prosthetic applications.

Yue et al. developed a polyaniline hybrid hydrogel using the precursors acrylic acid, ethylene oxide, and aniline [107]. The synthesized hydrogel demonstrated significant stretchability and great fatigue resistance owing to the electrostatic interaction and reversible hydrogen bonds among polyacrylic acid/polyethylene oxide and polyaniline networks. The reactive shaping method enabled the 3D printing of the hydrogel into prefabricated microlattice structures that have dual-conducting electron/ion routes and great elasticity. This makes the hydrogel a potential material for stretchy conductors in capacitive pressure sensors. It showed a broad detecting range and a high sensitivity of 7.10 kPa^−1^, making it competent for applications in physiological signal detections, prosthetic E-skin, and human–machine interfaces. 

### 5.3. Flexible pH Sensors

**Figure 10:**
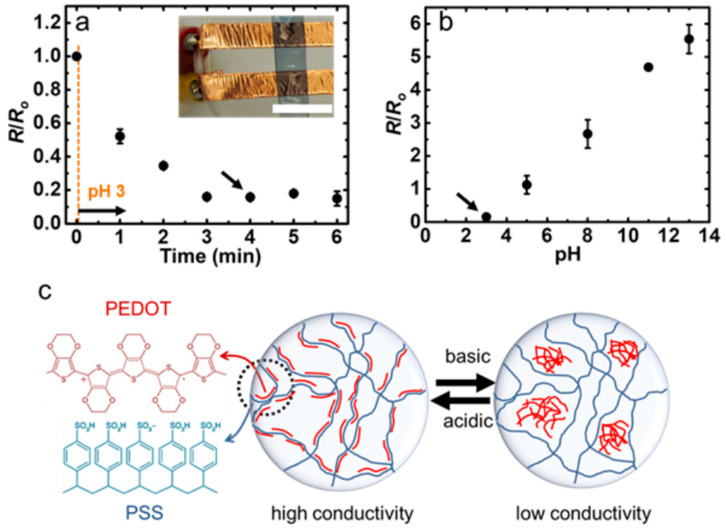
The electrical response of the hydrogel sensor to the pH change from neutral to pH 3 (**a**), the correlation between resistance and pH of the solution for the hydrogel sensor (**b**), The arrows in (**a**,**b**) indicate the same data points. Schematic representation of the influence of pH on the molecular structure of hydrogel sensor (**c**). Reproduced with permission [108]. Copyright © 2018 WILEY-VCH Verlag GmbH & Co. KGaA, Weinheim.

pH is regarded as a significant environmental signal, offering substantial insights into the surrounding environment. Being a measure of the solution’s proton activity, pH directly correlates with other environmental signals like the CO_2_ concentration in aqueous solutions or the activity of specific biological species. Traditional methods for pH determination, such as spectrometry or electrochemistry, often involve inflexible materials that are incompatible with flexible systems. Hydrogel-based pH sensors hold a greater promise as demonstrated by a study where poly(3,4-ethylenedioxythiophene) was combined with polyurethane to develop new printed inks with biomechanical characteristics [108]. These inks were exploited for the 3D extrusion printing of flexible hydrogel pH sensors capable of linearly responding to pH changes in a wet environment. The competence of this pH sensor was quantified by dipping it in solutions of varied pH both in ascending order (3–11) and descending order (11–3). The sensor depicted an electrical response of 200 μm when the medium switched from Milli-Q water to pH 3 (Figure 10a). The resistance changed almost linearly in both scenarios with pH variations (Figure 10b), and the sensor was functional for two months while stored in Milli-Q water. Furthermore, the ionic interaction between different chains in the sensor contributed to stabilizing the entire system (Figure 10c). 

Yin et al. employed optical maskless stereolithography to achieve high-precision printing of polyacrylic acid hydrogel onto an optical microfiber, leading to the creation of a flexible pH sensor [109]. The pH sensor depicted a sensitivity of 7.5 nm per pH, and the response times for the sensor for the rising and falling pH process were 100 and 130 s, respectively, indicating that the developed pH sensor has better sensitivity and swift response time in pH sensing. An electrically conductive hydrogel, based on polyethylene glycol diacrylate (PEGDA)-sulfonated polyaniline (PANI), was fabricated and printed via stereolithography to develop pH-sensing material [110]. The pH-responsive properties were evaluated within the pH range of 2.0 to 7.0, demonstrating a linear detection response across the studied range.

### 5.4. Flexible Temperature Sensors

**Figure 11:**
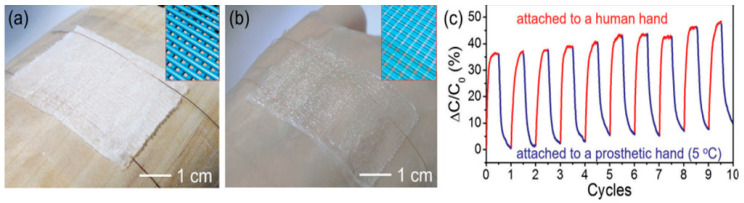
Photographs representing the thermo-responsive ionic skin attached to a prosthetic finger (**a**) and a human hand (**b**). Plots illustrating different grid structures at different temperatures (**c**). Reproduced with permission [113]. Copyright © 2017 Royal Society of Chemistry.

Human skin possesses the vital ability to sense temperature, which aids in illness diagnosis, accident prevention, and environmental awareness [111]. While conventional rigid temperature detectors like thermometers are practical, direct contact is ideal for accurate body temperature measurement, which is challenging with these rigid devices [112]. Therefore, the development of flexible temperature sensors is important. A thermo-responsive hydrogel was printed via 3D extrusion printing and serves as a temperature sensor capable of detecting body temperature, light finger touch, and bending motion owing to its sensitive and steady capacitance–temperature response and high pressure sensitivity within 1 kPa [113]. The flexible sensor exhibited excellent thermal sensing, undergoing a phase change at about 30 °C, transitioning from opaque (5 °C) to transparent on a human hand and a prosthetic hand (Figure 11a,b). Ten consecutive cycles between human and artificial hands maintained a relative capacitance change of approximately 37%, demonstrating reliable thermal response despite baseline drifting (Figure 11c). Liu et al. utilized the direct-ink-writing technique to fabricate a stretchable temperature sensor [114]. The sensitivity of this sensor was 0.008 °C^−1^, which is double that of commercial platinum sensors, demonstrating the remarkable temperature-sensing capabilities of the flexible sensor. Huang et al. used direct-ink-writing technology to produce economical, structure-specific, and situation-relevant MXene-based hydrogel sensors with exceptional temperature-sensing capabilities [115]. This sensor exhibited a GF (5.7) at 0–191% strain and temperature sensitivity of −5.27% °C^−1^ within a range (0–80 °C). To check the temperature sensitivity, copper electrodes were fastened to the hydrogel to assess efficient electron transfer in temperature-altering environments, revealing a positive temperature response in conductivity. The measured resistance demonstrated exceptional temperature sensitivity, decreasing from 4.36 MΩ at 0 °C to 0.093 MΩ at 80 °C.

### 5.5. Flexible Biosensors

Biosensors are analytical tools with the ability to integrate a biological element with a physicochemical sensor to transform a biological reaction into an electrical, optical, or measurable signal. These devices are created to identify biomolecules, typically with great sensitivity and precision [116]. Hydrogels with biocompatibility could be potentially fabricated by 3D-printing technology and employed as competent biosensors. A biocompatible hydrogel network based on p(HEMA-co-EGMA)/PEDOT: PSS was fabricated via SLA with a compressive modulus of 80 kPa and neural progenitor PC-12 cells and demonstrated a competent utilization as a biosensor in neural tissue engineering [117]. A study reported the fabrication of 3D-printed hybrid hydrogel consisting of PEO/PEDOT: PSS and PLLA nanofibrous mesh. The material confirmed a promising usage in biodevice production with a conductive coating [118]. A study demonstrated the amalgamation of chitosan-lactic acid with graphene oxide to design conductive hydrogels. The hydrogel was facilely processed via DIW into 3D scaffolds with fibroblast cells showing strong adherence and proliferation on the surfaces of the hydrogels. The hydrogel exhibited tunable conductivity and tensile strength in the range of 0.01–15.00 µS m^−1^ and 272–372 kPa. Further, it depicted good biocompatibility with L929 mouse fibroblast cells and water swellability of 110–260% [119]. An electrochemical biosensor developed via inkjet printing of polyaniline hydrogels revealed a rapid response time (0.3 s) along with higher sensitivity (16.7 µA mm^−1^) [120]. 

**Table 1:** Summary of 3D-printed flexible hydrogels for sensing applications.

Hydrogels	Printing Techniques	Application	Ref.
Poly(ACMO)/Pt	DLP	Strain sensor	[91]
κ-Carrageenan/PAAm	DIW	Strain sensor	[92]
B-PVA/kC	Inkjet	Strain sensor	[93]
Pul-SH/PDA/MoS_2_	Extrusion	E-skin	[95]
Ca-PAA-SA-CNTs	Extrusion	E-skin	[96]
H/G4	DIW	Glucose sensor	[102]
MRDN	Extrusion	Pressure sensor	[105]
AA/Aam/MgCl_2_	DLP	Pressure sensor	[106]
Poly(3,4-ethylenedioxythiophene/PU	Extrusion	pH sensor	[108]
Poly(acrylic acid)	DLP	pH sensor	[109]
(PEGDA)/sulfonated PANIs	DLP	pH sensor	[110]
Microstructured hydrogels	Extrusion	Temperature sensor	[113]
Graphene/Polydimethylsiloxane	DIW	Temperature sensor	[114]
MXene hybrid hydrogel	DIW	Temperature sensor	[115]
p(HEMA-co-EGMA)/PEDOT: PSS	SLA	Biosensor	[117]
Chitosan-PLA-GO	DIW	Biosensor	[119]

## 6. Conclusions

In summary, we provide an overview of recent advancements in the realm of flexible hydrogel sensors produced through 3D-printing technology. The initial focus lies in understanding the inherent characteristics of hydrogels, which govern the applications of flexible sensors. Hydrogels are classified into ion-type and electron-type, with the latter requiring free-moving electrons for electrical conductivity and the former relying on sufficient free ions to establish an ionic pathway. The discussion delves into the debate surrounding using 3D-printing technology in crafting hydrogel-based flexible sensors. Additionally, we extensively review the applicability of 3D-printed flexible hydrogel sensors as strain, pressure, pH, temperature, and biosensors, elucidating the underlying sensing mechanisms. Hydrogel-based flexible sensors have come a long way over the decades, but there are still several unsettled obstacles standing in the way of their harmonious integration into our daily lives. 

## 7. Perspectives

The amalgamation of hydrogels and 3D printing is a viable approach for the creation of intricate structures and meticulous material modification, all while lowering production costs, but there are still various flaws to address before these flexible sensors become relevant at a larger scale. 

(a) The issue of simultaneous optimization of electrical conductivity and mechanical strength is still prevalent as interface effects arise between the fillers and the hydrogel on the addition of nanofillers, compromising the mechanical strength. Hydrogels, owing to their innate properties, restrict the range of 3D-printing processes that are available.

(b) The discrepancy in physical and mechanical properties at the boundary between hydrogel sensors and microelectronic devices is a long-standing problem and thus the incorporation of flexible hydrogel sensors and device modules is imperative to be addressed.

(c) The prerequisite of the laser-based printing method is the photocurable features of the material; high shear and thixotropic characteristics of hydrogels are imperative for extrusion-based printing; and low-viscosity hydrogels are fitting for inkjet-based printing. To achieve the performance criteria in real-world applications, hydrogel materials’ sensitivity and stability must be significantly improved. For maintaining stable monitoring over an extended period, it is also indispensable to address the longevity and stability challenges of the hydrogel materials.

(d) The conductivity and incessant stability of 3D-printed hydrogels in terms of their mechanical and electrical properties must be upscaled to be used in real-world situations as electrodes in triboelectric and capacitive strain sensors.

(e) Dehydration of hydrogels results in decreased electrical conductivity and mechanical properties, impacting their sensing capabilities and causing insensitivity. Adding a binary solvent like glycerin has been used to enhance the water retention ability of the hydrogel to withstand extreme temperatures, but it can impede polymerization and result in reduced sensing capabilities. Thus, it is imperative to find a solution to limit the dehydration of hydrogels to maintain the sensing ability. 

(f) Additional research should be carried out into creating 3D-printed hydrogels using bioinspired polymers in an attempt to advance the biocompatibility, biodegradability, and disposability of hydrogel-based sensors.
